# Glutathione-*S*-transferase M1, T1 and P1 polymorphisms, and breast cancer risk, in BRCA1/2 mutation carriers

**DOI:** 10.1038/sj.bjc.6604394

**Published:** 2008-05-27

**Authors:** L Kadouri, Z Kote-Jarai, A Hubert, M Baras, D Abeliovich, T Hamburger, T Peretz, R A Eeles

**Affiliations:** 1 Sharett Institute of Oncology, Hebrew University-Hadassah Medical Center, Jerusalem, Israel; 2 Cancer Research UK Section of Cancer Genetics, The Institute of Cancer Research, Sutton, UK; 3 Braun School of Public Health, Hebrew University-Hadassah Medical Center, Jerusalem, Israel; 4 Human Genetic Laboratories, Hebrew University-Hadassah Medical Center, Jerusalem, Israel; 5 The Royal Marsden NHS Foundation Trust, Sutton, UK

**Keywords:** breast cancer, BRCA1/2, glutathione-*S*-transferase, GSTP1 polymorphism, modifiers

## Abstract

Variation in penetrance estimates for BRCA1/2 carriers suggests that other environmental and genetic factors may modify cancer risk in carriers. The GSTM1, T1 and P1 isoenzymes are involved in metabolism of environmental carcinogens. The *GSTM1* and *GSTT1* gene is absent in a substantial proportion of the population. In *GSTP1*, a single-nucleotide polymorphism that translates to *Ile112Val* was associated with lower activity. We studied the effect of these polymorphisms on breast cancer (BC) risk in *BRCA1/2* carriers. A population of 320 *BRCA1/2* carriers were genotyped; of them 262 were carriers of one of the three Ashkenazi founder mutations. Two hundred and eleven were affected with BC (20 also with ovarian cancer (OC)) and 109 were unaffected with BC (39 of them had OC). Risk analyses were conducted using Cox proportional hazard models adjusted for origin (Ashkenazi *vs* non-Ashkenazi). We found an estimated BC HR of 0.89 (95% CI 0.65–1.12, *P*=0.25) and 1.11 (95% CI 0.81–1.52, *P*=0.53) for the null alleles of *GSTM1* and *GSTT1*, respectively. For *GSTP1*, HR for BC was 1.36 (95% CI 1.02–1.81, *P*=0.04) for individuals with *Ile/Val*, and 2.00 (95% CI 1.18–3.38) for carriers of the *Val/Val* genotype (*P*=0.01). An HR of 3.20 (95% CI 1.26–8.09, *P*=0.01), and younger age at BC onset (*P*=0.2), were found among *Val/Val, BRCA2* carriers, but not among *BRCA1* carriers. In conclusion, our results indicate significantly elevated risk for BC in carriers of *BRCA2* mutations with *GSTP1-Val* allele with dosage effect, as implicated by higher risk in homozygous *Val* carriers. The *GSTM1*- and *GSTT1*-null allele did not seem to have a major effect.

The variability of cancer risk among *BRCA1/2* carriers (Struewing *et al*, 1997; Ford *et al*, 1998; Thorlacius *et al*, 1998; Antoniou *et al*, 2003) suggests a role for environmental and genetic modifiers. The impaired DNA-repair mechanism in carriers of *BRCA1/2* mutation (Boulton, 2006) may result in higher vulnerability to reduced activity of other genes that participate in maintaining genome stability. The glutathione-*S*-transferase superfamily of enzymes participates in protection against exogenous and endogenous oxidative damage (Hayes and Pulford, 1995). They conjugate xenobiotics, such as herbicides, insecticides and other environmental carcinogens, and anticancer agents (alkylating agents, platinum compounds) to glutathione to facilitate excretion. In addition, endogenous electrophile molecules produced through metabolism of lipid and DNA products of oxidative stress, as well as oxidative metabolites of oestrogen, the catechol oestrogen, are detoxified by these enzymes (Cavalieri *et al*, 2000; Dawling *et al*, 2004).

Homozygous absence of both alleles coding for the *GSTM1* (Seidegard *et al*, 1985) and *GSTT*1 (Pemble *et al*, 1994) are commonly found in various populations (50 and 20% respectively; Rebbeck, 1997). Elevated DNA adducts, sister-chromatid exchange and somatic genetic mutation have been demonstrated in carriers of null *GSTM1* and *GSTT*1 genotypes (Rebbeck, 1997). In *GSTP1* gene, a polymorphism of A315G encodes substitution of the wild-type isoleucine to valine at position 105 (*Ile*105*Val*). The valine variant was reported to have a reduced activity when recombinantly expressed in *Escherichia coli* (Zimniak *et al*, 1994).

The role of these genes in breast cancer (BC) has been evaluated through numerous case–control studies yielding conflicting results. In a meta-analysis in 1999 (Dunning *et al*, 1999), the *GSTP1 Ile/Val* genotype had odds ratio (OR) for BC of 1.6 (*P*=0.02). *GSTM1* was significantly associated with postmenopausal BC and for *GSTT1*, a moderate effect of 1.5 risk elevation could not be excluded. A recent large case–control study in Shanghai observed an elevated BC risk of approximately 2 among the *GSTP1*-*val* homozygotes, which was similar among pre- and postmenopausal women (Egan *et al*, 2004). The authors also reported an updated meta-analysis based mainly on studies in the Caucasian population, which supports null results for these three polymorphisms. A subsequent pooled analysis based on approximately 2000 cases and controls was also negative for the three *GST* polymorphisms (Vogl *et al*, 2004).

In this study, we report the effect of these polymorphisms on BC risk and age at onset among *BRCA1/2* carriers (Figures 1 and 2).

## METHODS

### Subjects and methods

#### Study population

The study population has been reported previously (Kadouri *et al*, 2004a). In summary, blood samples from 320 *BRCA1/2* carriers were collected through two centres: 240 carriers were identified by the oncology department and the cancer genetic clinic in the Hadassah Medical Centre in Jerusalem, Israel, and 80 at the cancer genetic carrier clinic in the Royal Marsden NHS Foundation Trust, London, UK. Cases were tested on the basis of a family history of BC and/or ovarian cancer (OC), or on the basis of their Ashkenazi origin. All the cases from Jerusalem were of Ashkenazi origin; of them all, but one, carried one of the three Ashkenazi founder mutations (132 cases: 185delAG, 32 cases: 5382insC in *BRCA1* and 75 cases: 6174delT in *BRCA2*, one carried other *BRCA2* mutations). The carriers from UK included 23 carriers of the Ashkenazi founder mutations (12 cases: 185delAG, 5 cases: 5382insC and 5 cases: 6174delT and two carriers of both 185delAG and 6174delT) and 56 carriers of other mutations (47 *BRCA1*; 9 *BRCA2*, specific mutation have been reported elsewhere; Kadouri *et al*, 2004b). Of the 320 carriers, 191 were affected with BC, 39 with OC and 20 with both cancers. Seventy of the mutation carriers were unaffected.

### Genotyping

DNA was salt-extracted from blood samples by standard methods. *GSTM1* and *GSTT1* genotypes were determined by PCR amplification and agarose-gel electrophoresis. The *INFA5* (5′--ggcacaacaggtagtaggcg--3′, 5′-gccacaggagcttctgacac-3′) gene was used as an internal control; this method conclusively identifies the null genotypes (homozygous deletion of the gene). Homozygous non-deleted and heterozygous genotypes were not distinguished from each other. The *GSTP1* (*Ile*105*Val*) genotypes were determined by PCR–RFLP. For all PCR reactions, 25 ng of genomic DNA was used in a 15-μl reaction mixture containing 1.5 mM MgCl_2_, 6 pM of each primer, 0.5 U of Amplitaq Gold polymerase (The Perkin Elmer Corp. Norwalk, CT, USA) and were run in a Hybaid Touchdown PCR machine. Primers were as follows: *GSTM1*, 5′--ctgccctacttgattgatggg--3′, 5′--ctggattgtagcagatcatgc--3′; *GSTT1*, 5′--ttccttactggtcctcacatctc--3′, 5′--tcaccggatcatggccagca--3′; *GSTP1*: 5′--acccagggctctatggggaa--3′, 5′--tgagggcacaagaagcccct--3′. Annealing temperature was 55°C for *GSTM1* and *GSTP1*, and 66°C for *GSTT1*. Assignment of the *GSTP1 Ile* (ACA TCT) and *Val* (ACG TCT) genotype was made by digestion of the PCR products on the basis of the RFLP with *Bsm*AI (New England Biolabs, Hertfordshire, UK). All PCR products were separated on 3% agarose gels with 2 μl/100 ml of ethidium bromide. Affected and unaffected samples were randomly located in the plates, a control sample was localised in each plate and five of the samples were genotyped twice with 100% concordance for the three genes. Call rates were high with one sample that could not be genotyped at all and an additional failure in *GSTT1* and *GSTP1* genotypes.

### Statistical analysis

The effects of *GST* genotypes on BC risk in mutation carriers were evaluated using a COX proportional hazards model. Participants were followed up retrospectively from date of birth to several possible outcomes. The outcome in women affected with BC was recorded as the age at first BC diagnosis. Women unaffected with BC were censored at the date of OC diagnosis, prophylactic surgery, date of last follow upor death. Since distributions of age and disease status were different in the Ashkenazi and non-Ashkenazi populations, the analyses were adjusted for ethnic origin. Although selection of participants is partly based on outcome, this method of analysis was used previously for risk estimation in carriers (Rebbeck *et al*, 1999; Levy-Lahad *et al*, 2001; Kadouri *et al*, 2004a). In our study (Kadouri *et al*, 2001) on the modifying effect of the androgen receptor-CAG-repeat length in *BRCA* carriers, we compared COX proportional hazard models to a variant of the log rank designed to overcome selection bias by comparison of outcome to expected penetrance according to the literature. Since estimated risks were almost similar in both methods, in the current paper we have used COX proportional hazards models. The *GSTM1* and *GSTT1* genotypes were classified as either null (i.e., homozygous deletion) or non-deleted (i.e., heterozygous or homozygous for the presence of the gene). For *GSTP1* polymorphism, where there were more than two genotypes, ORs were compared with the more common genotype, the *Ile* homozygous genotype. Comparison of ages at BC onset were calculated by ANOVA. We adjusted the *P*-values for multiple comparisons following HOLM's procedure both for pairwise comparisons of origin (Ashkenazi/non-Ashkenazi) with the *GST* genotypes and for the COX regression models as shown in Table 1.

## RESULTS

The frequency of the *GSTM1*- and *GSTT1*-null and *GSTP1* alleles in our Ashkenazi and non-Ashkenazi populations were as follows: *GSTM1* null in 52.1 *vs* 55.2% (*P*=0.77) and *GSTT1* null in 10.5 *vs* 26.6% (*P*=0.03), respectively. *GSTP1 Ile/Ile, Ile/Val* and *Val/Val* were found in 64.6, 30.4 and 4.9% of the Ashkenazi population, compared with 49.1, 40.4 and 10.5% in non-Ashkenazi, respectively (*P*=0.03), and all were in Hardy–Weinberg in both populations.

Table 1 shows the frequencies of the *GSTM1*, *GSTT1* and *GSTP1* genotypes in *BRCA1/2* carriers both with and without BC, and the corresponding hazard ratios (HR). The *GSTM1*- and *GSTT1*-null genotypes were not associated with BC risk. Frequency of the *GSTM1*-null allele was non-significantly lower in BC cases (49.8%) than in BC-free *BRCA1/2* mutation carriers (57.8%), and the estimated HR was 0.89 (95% CI 0.65–1.12, *P*=0.25). The corresponding frequencies for the *GSTT1*-null allele were 25.1 and 22.2% in BC cases and in BC-free carriers with a non-significant HR of 1.11 (95% CI 0.81–1.52, *P*=0.53). There was, however, evidence of increasing BC risk with increasing number of *GSTP1*-*Val* alleles; the HR for developing BC was 1.36 (95% CI 1.02–1.81, *P*=0.04) and 2.00 (95% CI 1.18–3.38, *P*=0.01), respectively, for *Ile/Val* heterozygotes and *Val/Val* homozygotes, as compared with that for *Ile/Ile* homozygotes (see *P*-values after correction for multiple comparisons in the footnote of Table 1).

**Figure 1:**
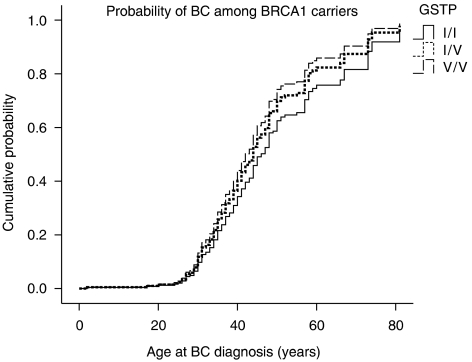
Probabilities of BC with age according to *GSTP1* genotypes (*Ile/Ile*, *Ile/Val*, *Val/Val*) among *BRCA1* carriers (analyses were adjusted to origin, Ashkenazi *vs* non-Ashkenazi).

**Figure 2:**
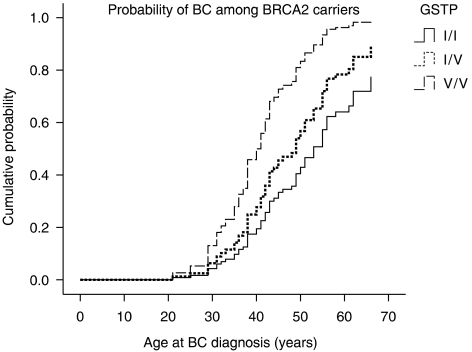
Probabilities of BC with age according to *GSTP1* genotypes (*Ile/Ile*, *Ile/Val*, *Val/Val*) among *BRCA2* carriers (analyses were adjusted to origin, Ashkenazi *vs* non-Ashkenazi).

In a separate analysis for the role of *GSTP1* genotypes in *BRCA1* and *BRCA2* carriers (Table 2, Figure 2), a significant effect was found among the *BRCA2* carriers. The HR for developing BC was 1.50 (95% CI 0.86–2.59, *P*=0.6) and 3.20 (95% CI 1.26–8.09, *P*=0.01), respectively, for *Ile/Val* heterozygotes and *Val/Val* homozygotes, as compared with that for *Ile/Ile* homozygotes. Younger mean age of 41.2 years at BC onset was found among *Val/Val* homozygotes as compared with 46.6 years in *Ile/Ile* homozygotes (*P*=0.2). In *BRCA1* carriers, the effect of the *GSTP1*-*Val* allele was non-significant (Table 2, Figure 1).

## DISCUSSION

Our results do not show a major effect of the null genotypes of the *GSTM1* and *GSTT1* on BC risk in *BRCA1/2* mutation carriers. However, the *GSTP1*-*Val/Val* allele was associated with approximately twofold BC risk in *BRCA1/2* carriers. In *BRCA2* carriers, a significant HR of 3.2 was found in homozgotes for the *Val* allele, whereas the effect among *BRCA1* carriers was non-significant. To our knowledge this is the first report of an effect of *GST* polymorphism in *BRCA1/2* carriers. A major limitation of our study is the survival bias due to inclusion of individuals while alive. This is an important consideration also regarding previous genetic risk modifiers reported among *BRCA1/2* carriers, since the effect of these modifiers on survival is unknown. The effect of the *GST*-*Val* allele on BC prognosis contradicted in previous studies (Goode *et al*, 2002; Yang *et al*, 2005). Therefore, larger studies based on incident cases are warranted.

The GSTP1 isoenzyme is highly expressed in the mammary epithelium, both in normal and in tumor cells (Forrester *et al*, 1990; Kelley *et al*, 1994). It has been shown that methylation of the promoter (Arai *et al*, 2006) and low expression of the *GSTP1* gene is associated with poor prognosis in BC patients treated with chemotherapy (Arai *et al*, 2006). In addition to its role in detoxifying electrophilic molecules from exogenous exposures, the *GSTP1* gene has an important role in the metabolism of estradiol derivatives (Cavalieri *et al*, 2000; Dawling *et al*, 2004). It reduces the concentration of oestrogen quinones, thereby reducing the potential of these oxidative oestrogen metabolites to induce DNA damage. It is possible that in cells, which are deficient in functional BRCA1 or BRCA2, key proteins in DNA-damage repair, the DNA will be vulnerable to oxidative damage. The role of the BRCA2 protein in DNA double-strand break (DSB) repair is well established (Boulton, 2006). The BRCA2 protein has a distal role in the DNA-repair machinery (Boulton, 2006). It forms a complex with the RAD51 protein, which is essential for DNA repair through homologous recombination (Davies *et al*, 2001). However, the direct function of BRCA1 in DSB repair is less clear, although a more proximal role in sensing and regulation of cellular response to DNA damage has been suggested by several recent papers (Boulton, 2006). More importantly, BRCA1 upregulates the expression of genes involved in antioxidant response, including *GST* genes (Bae *et al*, 2004). In accordance, BRCA1 deficiency conferred sensitivity to several oxidising agents in cell lines (Bae *et al*, 2004). The lack of effect in *BRCA1* carriers could be related to the already high oxidative stress in cells deficient in the BRCA1 protein; the addition of low active GSTP1 does not significantly add to the process of tumorigenesis. However in *BRCA2* carriers, low level of active GSTP1 results in higher DNA damage properly sensed by BRCA1, but ineffectively corrected by the BRCA2 complex.

Several modifier genes were reported in carriers of *BRCA1/2* mutation. A modifying effect has been confirmed by two or more separate studies for two of these modifiers. Interestingly, in both genes, the effect differed among *BRCA1* and *BRCA2* carriers. A rare polymorphism in the *RAD51* gene was associated with BC risk in *BRCA2* carriers (Levy-Lahad *et al*, 2001; Wang *et al*, 2001; Kadouri *et al*, 2004a). Recently, a large study among approximately 8500 mutation carriers confirmed a modifying effect for the homozygous, but not the heterozygous, *RAD51* 135 g/c polymorphism (Antoniou *et al*, 2007). On the other hand, a polymorphic CAG-repeat length in the *AIB1*, a coactivator of the oestrogen receptor, was found to modify BC risk in *BRCA1* carriers (Rebbeck *et al*, 2001; Kadouri *et al*, 2004b). Two recent, large studies did not observe a modifying effect for the *AIB1*-CAG repeat among *BRCA1/2* carriers (Hughes *et al*, 2005; Spurdle *et al*, 2006). It is possible that these larger studies included heterogeneous population both genetically and clinically; therefore, association may be either lost or exaggerated. Indeed, distributions of three polymorphisms previously reported by us, the *AR*-CAG repeat (Kadouri *et al*, 2001), *RAD51*-g/c-SNP (Kadouri *et al*, 2004a) and the *AIB1*-polyglutamine chain length (Kadouri *et al*, 2004b), as well as the GSTP1 in the current study, were significantly different among Ashkenazi Jews as compared with British Caucasian women. Since we included two distinct populations, we were able to effectively adjust for origin.

In conclusion, in the present study we report a modifying effect of the *GSTP1* gene in *BRCA2* but not *BRCA1* carriers. Carriers of homozygous *Val/Val* allele, a less active variant of the GSTP1 protein, had an approximately threefold risk for BC and younger age at onset as compared with *Ile/Ile* carriers. This adds to the mounting evidence suggesting differences in molecular mechanisms involved in normal function and tumor formation in *BRCA1* and *BRCA2* carriers. These positive results obtained from a small population of *BRCA2* carriers should prompt a larger study in other populations as well.

## Figures and Tables

**Table 1:** *GSTM1*, *T1* and *P1* allele frequencies and BC HR in *BRCA1/2* carriers (adjusted for origin, Ashkenazi *vs* non-Ashkenazi)

**Genotype**	**BC− (*n*=109) no. (%)**	**BC+ (*n*=211) no. (%)**	**Average age at BC onset (years)**	**Breast cancer HR (95% CI)**	***P*-value**
***GSTM1*** *(n=320)*					
Present	46 (42.2)	106 (50.2)	41.4	1	
Null	63 (57.8)	105 (49.8)	42.9	0.89 (0.65–1.12)	0.25^a^
***GSTT1*** *(n=319)*					
Present	84 (77.8)	158 (74.9)	42.5	1	
Null	24 (22.2)	53 (25.1)	41.3	1.11 (0.81–1.52)	0.53^b^
***GSTP1*** *(n=319)*					
*Ile/Ile*	76 (70.6)	121 (57.3)	42.1	1	
*Ile/Val*	29 (26.6)	74 (35.1)	42.6	1.36 (1.02–1.81)	0.04^c^
*Val/Val*	3 (2.8)	16 (7.6)	40.5	2.00 (1.18–3.38)	0.01^d^

BC−, no BC; BC+, with BC; CI, confidence interval; HI, hazard ratio.

Corresponding *P*-values after correction for multiple comparisons are as follows: ^a^0.50, ^b^0.53, ^c^0.12 and ^d^0.03.

**Table 2:** *GSTP1* allele frequencies and BC HR in *BRCA1* and *BRCA2* carriers (adjusted for origin, Ashkenazi *vs* non-Ashkenazi)^a^

	***BRCA1* (*n*=228)**			***BRCA2* (*n*=90)**		
***GSTP1***	**BC− (*n*=78) no. (%)**	**BC+ (*n*=150) no. (%)**	**Breast cancer HR (95% CI, *P*)**	**BC− (*n*=29) no. (%)**	**BC+ (*n*=61) no. (%)**	**Breast cancer HR (95% CI, *P*)**
*Ile/Ile*	53 (67.9)	86 (57.3)	1	22 (75.9)	35 (57.4)	1
Age at BC		40.5 years			46.6 years	
						
*Ile/Val*	22 (28.2)	54 (36)	1.22 (0.87–1.72, *P*=0.24)	7 (24.1)	20 (32.8)	1.50 (0.86–2.59, *P*=0.15)
Age at BC		42.6 years			42.6 years	
						
*Val/Val*	3 (3.9)	10 (6.7)	1.38 (0.71–2.70, *P*=0.15)	0	6 (9.8)	3.20 (1.26–8.09, *P*=0.01)
Age at BC		40.1 years			41.2 years	

BC−, no BC; BC+, with BC; CI, confidence interval; HI, hazard ratio.

^a^Compound heterozygotes were excluded from these analyses.
